# Massive paediatric cervicofacial actinomycoses masquerading as an ulcerative malignancy

**DOI:** 10.1186/s12879-016-1768-8

**Published:** 2016-08-15

**Authors:** Carlson–Babila Sama, Nicole Fouda Mbarga, Calvin Eta Oben, Jules A. Mbarga, Elvis Kiloh Nfor, Fru F. Angwafo III

**Affiliations:** 1 Islamic Medicalised Health Centre, Babessi, Cameroon and Galactic Corps Research Group (GCRG), Buea, Cameroon; 2 SporeData Inc., Durham, United States of America and Sangmelima District Hospital, South Region, Cameroon; 3 Community Humanitarian Emergency Board, Bangui, Central African Republic; 4 Croix Rouge–Française, Yaoundé, Cameroon; 5 Djohong District Hospital, Adamawa Region, Cameroon; 6 Gynaeco-Obstetric and Paediatric Hospital, Yaoundé, Cameroon

**Keywords:** Cervicofacial, Actinomycosis, Malignancy, Cameroon

## Abstract

**Background:**

Paediatric cervicofacial actinomycosis is a rare infectious disease caused by *Actinomyces spp.* and usually presents as a chronic, suppurative and granulomatous inflammation with a propensity to mimic malignant conditions.

**Case presentation:**

We discuss the case of an 11-year-old African female who presented with a chronic disfiguring cervical mass evolving over a 9 months period for which she had several unyielding consultations. Appropriate clinical and para-clinical evaluations were paramount to the diagnosis of an *Actinomyces* infection. We review the literature on its epidemiology, clinical presentation, diagnosis, treatment and prognosis.

**Conclusion:**

Actinomycosis still poses a diagnostic challenge. It is important for clinicians to consider the possibility of such rare infections in apparently malignant looking masses and also in lesions not responding to several antimicrobial treatments. The condition generally carries a good prognosis if recognised early and histopathological diagnosis is the gold standard.

## Background

Actinomycosis (AM) is a rare, slowly progressive, chronic granulomatous inflammatory disease that may result in multiple abscesses, draining sinus tracts, tissue fibrosis and fistulae formation [1, 2]. It is caused by a group of gram-positive, filamentous, anaerobic bacilli of the *Actinomyces* genus which are endogenous microbiota of the mouth, gastrointestinal and genitourinary tracts [2]. These microorganisms are usually commensals; hence disruption of mucosal integrity and devitalised tissue are necessary for invasion of subcutaneous planes. It usually spreads contiguously to adjacent soft tissues, ignoring tissue planes and lymphatic drainage [3, 4]. Cervicofacial AM is the most common presentation of the disease and dental infections or extraction and maxillofacial trauma are predisposing factors. It often evolves as a subacute or chronic soft tissue swelling of the submandibular or paramandibular regions [3–6].

The diagnosis of AM can easily be missed because it has the tendency to mimic a number of other conditions including malignancies and granulomatous diseases [4, 7–9]. More so, *Actinomyces spp* are very susceptible to a wide variety of antimicrobials, thus relatively few doses may render cultures negative [6]. However, its proper diagnosis is of prime importance due to the fact that AM can be disfiguring or even fatal if vital structures including airways and major vessels are involved. Furthermore, it requires a remarkably long duration of treatment for its complete eradication [3, 6, 10]. We herein report, possibly the first case of cervicofacial actinomycosis from Cameroon and a brief review of literature on the topic.

## Case presentation

The patient is an 11-year old sub-Saharan African girl who presented with chief complains of pain and swelling in the neck. The swelling was first noticed 9 months earlier as a small, painless and mobile nodule over the left infra auricular region which has gradually increased in size over a period of 6 months to attain its current dimensions. During this time, the swelling became immobile, occasionally painful and developed a sinus tract which discharged thick, cream-white non-offensive pus. She had lost weight and was occasionally febrile but denied being anorexic or having night sweats during the course of her ailment. Review of hospital records showed she had been to several health institutions and received several doses of different classes of oral antibiotics and anti-inflammatory drugs with no definitive diagnosis. Usually, there was no significant relief of symptoms or improvement in general status. There was no history suggestive of an orodental infection or tooth extraction but she recalled a thorn prick injury to the left infra auricular region 2 weeks prior to onset of the swelling. Over the last 6 days, ulceration of the swollen tissue, increased pain and abundant malodorous purulent discharge prompted her parents to seek medical attention at our service. She is of a poor socioeconomic background and has no known chronic disease.

**Fig. 1:**
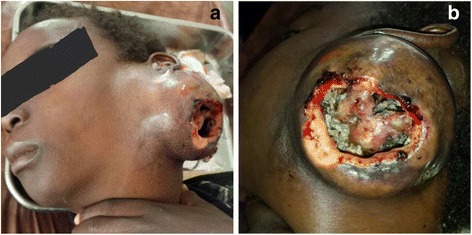
**a** Giant disfiguring cervicofacial mass. **b** Ulcerated mass containing yellowish pus

On examination, she looked frail, but vital signs were normal. There was a giant ulcerated 13 x 14 cm (cranio–caudal x transverse) disfiguring mass (Fig. 1a) at the left cervical region just below the left ear which contained yellowish pus (Fig. 1b). Tenderness, trismus and expansive hardness of the surrounding tissues were noted. There were no palpable lymph nodes. Apart from reduced mouth opening, intraoral examination was normal with good dental hygiene and no apparent congenital malformation. The rest of the examination was unremarkable. At this point, many differential diagnoses ranging from a tuberculous infection to neoplasms related to structures of the neck were considered. Laboratory investigations including full blood count and blood biochemistry were within normal values. HIV serology and Mantoux test were negative. Cervical x-ray ruled out bone involvement. Ultrasonography and computed tomographic scans were not done due to financial constraints. A wet mount preparation of pus sample demonstrated granules and gram stained smears showed gram–positive, filamentous bacteria. Aerobic culture of pus on blood agar and Mac Conkey agar were sterile after 24 hours of incubation at 37 °C. Pus samples were sent for anaerobic culture.

**Fig. 2:**
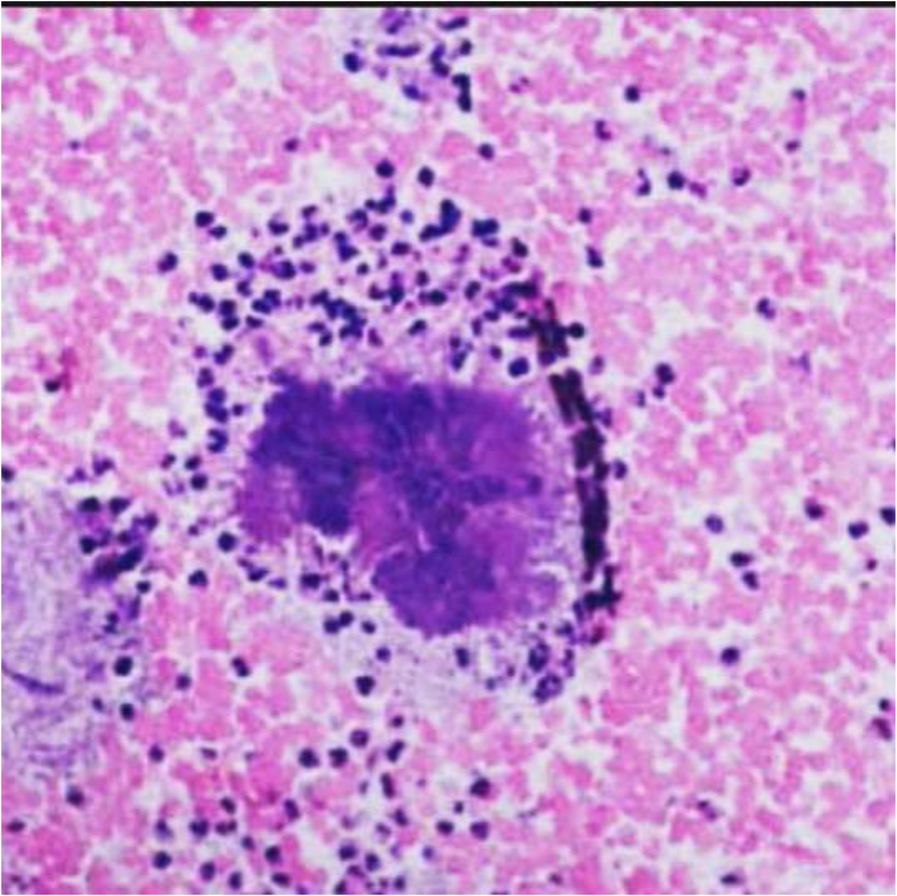
Haematoxylin–eosin stain showing Sulphur granules. The filaments show as a nondescript mass with heavy stain. Special stains are required to clearly demonstrate the filaments

Under general anaesthesia, multiple biopsy samples were obtained for histopathological diagnosis which showed no evidence of a neoplastic process. A Brown–Hopps tissue Gram stain revealed abundant gram–positive filamentous organisms consistent with *Actinomyces* (not further identified to species level) which also stained positively with Grocott-Gomori’s methenamin-silver nitrate staining. The histological examination revealed characteristic sulphur granules on a hematoxylin–eosin–stained section (Fig. 2). Zeihl–Neelsen staining for the presence of acid-fast bacilli (AFB) was negative. Based on the above findings, particularly the histopathological findings, we reached a diagnosis of cervicofacial actinomycoses. She was started on 20 million units of intravenous benzyl penicillin per day in 4 divided doses. Surgical debulking of the mass was delayed due to financial constraints. Two weeks into treatment, results from analyses of smears from anaerobic cultures on brain heart infusion blood agar in candle jar after 12 days of incubation at 37 °C were available. It revealed numerous *Actinomyces spp.,* a-hemolytic *Streptococcus, Leptotrichia buccalis* and *Lactobacillus spp.* Cultures for fungi were negative. Due to these findings, treatment with penicillin was switched to parenteral ertapenem. There was a remarkable improvement over a 4 week period with improvement in overall clinical status, decreased pain, significant reduction in size of the mass and the hard indurated surroundings became fluctuant and were surgically drained. Proper assessment of treatment outcome was hampered 6 weeks into treatment as patient did not return for follow-up and continuation of therapy.

## Discussion

The word Actinomycosis comes from the Greeks where “aktino” refers to the radiating appearance of sulphur granules and “mykos” refers to mycelia relating to a fungal disease. It is a rare infectious disease in humans but much more common in animals such as cattle, horses, pigs, and dogs [11]. In 1845, Von Langenbeck reported the first case in humans and attributed it to a fungus. In 1891, Israel and Wolff first isolated the microorganism from humans and Waksman showed that *Actinomyces* is a gram-positive bacterium; not a fungus in the 1960s [11, 12]. Over 30 species have been identified, of which the most common microorganism causing infections in humans is A. *israelii*, and to a lesser extent A. *naeslundii*, A. *meyeri*, A. *odontolyticus*, A. *viscosus*, A. *propionica*, and A. *gerencseriae* [2, 9, 11, 13].

These micro-organisms are filamentous, branched, gram-positive, anaerobic-to-microaerophilic, non-acid fast bacteria which are part of the resident microbiota of the oral cavity, gastrointestinal tract, and urogenital tract [2, 9, 11]. They are usually of low pathogenicity and in most cases, will cause disease only in the setting of poor oral hygiene, maxillofacial trauma, surgical procedures and dental manipulations leading to mucosal breakdown [12, 13]. In our patient, the portal of entry was thought to be a disruption of the mucosal barrier secondary to the thorn prick injury over the left cervical region.

The incidence of symptomatic AM infection is quite low and it is gradually decreasing [4]. It is an endemic infection occurring worldwide and there is no person to person transmission. Though not considered an opportunistic infection, it has been reported in various cases of immunocompromised hosts including HIV [14], leukaemia [15], diabetes [16] and use of immunosuppressive drugs [10]. However, majority of cases occur in immunocompetent individuals with no underlying disease. The occurrence of AM in a female paediatric age group as seen in our case is a rare entity as the disease generally has a peak incidence in the 4th to 6th decade of life with a slight male predominance and has no predilection for age, season, race, or occupation [9, 11, 17].

In 1938, Cope clinically classified AM infection into three distinct groups based on the affected site: cervicofacial, pulmonothoracic and abdominopelvic occurring in 50 %, 30 % and 20 % of cases respectively [11–13, 16]. The perimandibular region is the most common area to be affected in cervicofacial AM. It is also referred to as the “lumpy jaw disease” due to its characteristic woody, hard and board-like lumpy appearance when the jaws are affected as was observed in our patient [2, 10]. The incidence of AM infection affecting the various areas of the face are mandible (53.6 %), cheek (16.4 %), chin (13.3 %), submaxillary ramus and angle (10.7 %), maxilla (5.7 %) and temporomandibular joint (0.3 %) [11].

Cervicofacial AM classically presents as a firm, painless slow growing mass with surrounding hardening or erythema which progresses to fibrosis with or without suppuration. However, its clinical presentation may also be acute, with a rapidly progressive painful mass and purulent drainage from multiple sinus tracts discharging characteristic ‘sulphur granules’ [4, 11, 12]. These granules are rounded or elongated deep purple aggregates composed of filamentous organisms. They often have eosinophilic club–shaped ends and are often encrusted with protein in the Splendore–Hoeppli phenomenon [16]. Sulphur granules are present only in about 40 % of cases but their presence is pathognomonic [4]. Generally, Gram stains are more sensitive than cultures in aiding diagnosis, perhaps due to previous antibiotic use which may render the cultures negative or a lack of strict anaerobic processing [2, 10]. The infection usually spreads contiguously to adjacent soft tissues ignoring tissue planes and lymphatic drainage. Lymphadenopathy may develop due to secondary infection or late in the disease process [3, 4, 11, 12].

AM usually poses a diagnostic dilemma due to its enigmatic nature and high propensity to mimic a range of diseases including a neoplasm, tuberculosis, fungal infections, nocardiosis, and other chronic granulomatous conditions [4, 8–12, 16, 17]. In our case, a neoplasm was ruled out by histopathological studies; the possibility of a tuberculous infection became unlikely due to a negative Mantoux test. This was further supported by a non-detection of AFB which also ruled out norcardiosis which characteristically stains well on modified Zeihl-Neelsen. Furthermore, the diagnosis of AM becomes challenging due to a general lack of familiarity with the disease owing to its rarity and also a low success rate in culturing the organism as a result of its fastidious nature [12]. Actinomycosis is a masquerading infection suspected as the initial diagnosis in less than 10 % of documented cases [18, 19]. For these reasons, Acevedo et al. labelled it “a great pretender” [9] and it’s considered “the most misdiagnosed disease” [11]. It is also listed as a “rare disease” by the Office of Rare Disease of the National Institute of Health [11].

Imaging modalities such as computed tomographic scan, ultrasonography and magnetic resonance imaging usually yield non-specific features and are not contributory to a positive diagnosis. They may be helpful in guiding biopsy and also in delineating surgical margins, thus histopathological examination of biopsy specimen remains the gold standard for diagnosis [9, 11–13, 16].

Though our patient was lost to follow-up, penicillin G is the drug of choice in the treatment of AM and high doses (18 – 24 million units/day) over a 2 to 6 week period followed by oral therapy with penicillin or amoxicillin to complete a 6 to 12 month course has been proven effective in clinical experience rather than randomised controlled trials [3, 8, 9, 17]. However, the ultimate length of treatment will depend on the clinical and pathologic response. For penicillin-allergic patients, tetracycline, doxycycline, clindamycin, minocycline, erythromycin, and cephalosporins are effective options [9, 10]. As evidenced in the present case report, though *Actinomyces spp* was the dominant species, other pathogens coexisted, which is a common finding in perimandibular disease [2, 3, 10]. The polymicrobial nature of the infection prompted our use of ertapenem and it also has the convenience of once daily dosing. Importantly, several commonly used antimicrobials including the aminoglycosides, trimethoprim-sulfamethoxazole, metronidazole, aztreonam, cephalexin, penicillinase-resistant penicillins (e.g. oxacillin and nafcillin) are not active against *Actinomyces* species [1, 3, 10]. Surgery is an important adjunct and long term follow up is mandatory because relapse is common [4, 8, 11, 17]. If promptly recognised and treated, prognosis is excellent. Complete recovery is expected in over 90 % of patients with cervicofacial AM [18].

Mortality rate ranges from 0 to 28 % depending mainly on the site of infection and the time to diagnosis. Infections involving the central nervous system carries the greatest risk of mortality with deleterious neurologic sequelae reported in over half of these patients [9, 20].

## Conclusion

Cervicofacial AM is an uncommon disease that still poses a great diagnostic challenge to clinicians due to its insidious course, non-specific symptoms and a wide variety of clinical presentations. Therefore, clinicians should maintain a high index of suspicion and include it in the differential diagnosis of any soft tissue swelling of the head and neck. Our case also highlights the importance of collaborative efforts of clinicians in suspecting the disease and laboratory physicians in confirming it. Histopathological examination remains pivotal to its diagnosis. Delays in diagnosis and treatment may lead to chronic disfigurement, increased risk of morbidity and even mortality.

## Abbreviations

AFB: Acid-fast bacilli
AM: Actinomycosis
HIV: Human immunodeficiency virus
